# Assessment of Alertness and Cognitive Performance of Closed Circuit Rebreather Divers With the Critical Flicker Fusion Frequency Test in Arctic Diving Conditions

**DOI:** 10.3389/fphys.2021.722915

**Published:** 2021-08-10

**Authors:** Wilhelm W. Piispanen, Richard V. Lundell, Laura J. Tuominen, Anne K. Räisänen-Sokolowski

**Affiliations:** ^1^Faculty of Medicine, University of Turku, Turku, Finland; ^2^DAN Europe Research Division, Finnish Branch, Helsinki, Finland; ^3^The Centre for Military Medicine, The Finnish Defense Forces, Helsinki, Finland; ^4^Department of Pathology, Helsinki University, Helsinki, Finland; ^5^Anesthesia and Intensive Care Unit, Tampere University Hospital, Tampere, Finland; ^6^Department of Pathology, Helsinki University and Helsinki University Hospital, Helsinki, Finland

**Keywords:** inert gas narcosis, mixed gas diving, thermal control, technical diving, arctic diving

## Abstract

**Introduction:** Cold water imposes many risks to the diver. These risks include decompression illness, physical and cognitive impairment, and hypothermia. Cognitive impairment can be estimated using a critical flicker fusion frequency (CFFF) test, but this method has only been used in a few studies conducted in an open water environment. We studied the effect of the cold and a helium-containing mixed breathing gas on the cognition of closed circuit rebreather (CCR) divers.

**Materials and Methods:** Twenty-three divers performed an identical dive with controlled trimix gas with a CCR device in an ice-covered quarry. They assessed their thermal comfort at four time points during the dive. In addition, their skin temperature was measured at 5-min intervals throughout the dive. The divers performed the CFFF test before the dive, at target depth, and after the dive.

**Results:** A statistically significant increase of 111.7% in CFFF values was recorded during the dive compared to the pre-dive values (*p* < 0.0001). The values returned to the baseline after surfacing. There was a significant drop in the divers’ skin temperature of 0.48°C every 10 min during the dive (*p* < 0.001). The divers’ subjectively assessed thermal comfort also decreased during the dive (*p* = 0.01).

**Conclusion:** Our findings showed that neither extreme cold water nor helium-containing mixed breathing gas had any influence on the general CFFF profile described in the previous studies from warmer water and where divers used other breathing gases. We hypothesize that cold-water diving and helium-containing breathing gases do not in these diving conditions cause clinically relevant cerebral impairment. Therefore, we conclude that CCR diving in these conditions is safe from the perspective of alertness and cognitive performance.

## Introduction

It is known that diving in cold conditions imposes an increased risk of decompression sickness (DCI) for the diver (Gerth, 2015; Pendergast et al., 2015). It impairs physical and cognitive performance (Davis et al., 1975; Bridgman, 1990) and can lead to hypothermia or, if the exposure is prolonged, major health impairment or even death (Hayes, 1991). The diver’s body reacts to cold through peripheral vasoconstriction and diving responses, such as the trigeminocardiac reflex, lowering the heart rate (Konishi et al., 2016). These mechanisms impair the washout of nitrogen which can in turn affect the cognitive state of the diver. These changes can be monitored *via* a critical flicker fusion frequency (CFFF) measurement test.

The CFFF test is an acknowledged method for assessing subjects’ attention and alertness (Kahlbrock et al., 2012; Lu et al., 2012), which are influenced by the activation and depression of different parts of the central nervous system. The method has been used in various fields of medicine related to diving, spaceflight, internal medicine (Curran et al., 2004; Kowacs et al., 2005; Torlot et al., 2013; Barone et al., 2018), and psychology/toxicology. It has been found that stimulants increase CFFF and that alcohol, sedatives, and hypnotics decrease the value (Smith and Misiak, 1976; Hindmarch, 1982). It is an easy, noninvasive, objective, and relatively fast way of measuring attention and alertness (Lafère et al., 2010; Balestra et al., 2012, 2018; Hemelryck et al., 2013).

Most of the studies conducted in diving medicine in regard to CFFF have been conducted in pressure chambers or by breathing normobaric oxygen (NBO; Seki and Hugon, 1976; Hemelryck et al., 2013; Kot et al., 2015; Tikkinen et al., 2016; Lafère et al., 2019; Vrijdag et al., 2020) and only a select handful of studies have been done in pools or open water environments (Balestra et al., 2012; Lafère et al., 2016; Rocco et al., 2019). The results in these studies have varied or even been contradictory, most likely due to differences in the study set ups. In these studies, it has been found that the effect of oxygen on neuronal excitability is dose dependent, which has thought to be due to intertwined CFFF and oxygen poisoning transduction pathways (Kot et al., 2015). In fact, even NBO increases nerve conduction, this is probably related to reactive oxygen species (ROS; Brerro-Saby et al., 2010). This facilitation of nerve conduction has been shown to lead to better cognitive performance, such as memory, visuospatial, and verbal abilities (Moss and Scholey, 1996; Moss et al., 1998; Scholey et al., 1998, 1999; Chung et al., 2006). This excitation is thought to be a result of oxygen affecting GABA neurotransmission in multiple ways. Oxygen modulates the synthesis, secretion, and recapture of GABA (Radomski and Watson, 1973; Schwartz-Bloom and Sah, 2001).

Nitrogen and oxygen alone, or in combination, can produce neuronal excitability or depression in a dose-related response (Kot et al., 2015). There seems to be a complex competitive effect of both gases during hyperbaric exposure (Rocco et al., 2019), and that nitrogen narcosis does not dissipate instantly after surfacing (Balestra et al., 2012). It has been suggested that cold water may increase the narcosis phenomenon, but no objective proof has been unveiled on this topic. This may indicate that the narcosis phenomenon is more prevalent during uneventful dives than previously thought (Lafère et al., 2016). High nitrogen partial pressure (pN_2_) or a sudden increase in pN_2_, for example when rapidly descending during a dive, has been noted to be the most significant cause of inert gas narcosis (Bennett and Rostain, 2015). The exact mechanism and site of nitrogen narcosis is however still debated. One hypothesis for the mechanism of nitrogen narcosis is that pressure-dependent conformational changes occur due to gas-protein binding, particularly N-methyl-D-aspartate (NMDA) and gamma-aminobutyric acid (GABA) receptors in the substantia nigra (Rostain et al., 2011; Rostain and Lavoute, 2016). It is important to understand the factors affecting inert gas narcosis, since feared and possible effects of gas narcosis in diving include hallucinations and loss of consciousness. Therefore, it hampers the subject’s motor ability, and these factors could be detrimental to a diver’s own and their diving buddy’s life.

In this study, we aimed to examine, whether cold-water closed circuit rebreather (CCR) diving with helium-containing breathing gas would cause a different CFFF profile compared to results from earlier studies from warmer conditions and from divers using other breathing gases. Also, we aimed to examine if diving in our Arctic study conditions would cause changes in the diver’s cognitive function and alertness.

## Materials and Methods

### Subjects

The subjects were recruited from the Finnish technical diving community and were all experienced CCR divers. All divers participated voluntarily, and no financial benefit was received by participating in this study, but the expenses for the helium-nitrogen-oxygen gases used in these dives were compensated. All the divers filled out a health survey and a diving physician performed a fit-to-dive examination on the morning of the dives. All divers were fit to participate in the study. The demographics of the population are presented on Table 1. Twenty-four divers, 22 males, and two females of age (31–57 years) and experience level (300–1950 dives) participated in the tests. We excluded one diver from the analysis because his dry suit started to leak before the start of the dive, causing him to interrupt the test.

**Table 1:** Description of the population in the study.

Parameters	Average (*SD*, range), when applicable
Gender (Male/Female)	(21/2)
Age	44.9 (± 6.2, 31–57)
BMI (pre-dive)	27.6 (± 3.9, 23.4–39.8)
Fat% (pre-dive)	22.7 (± 7.7, 12.6–46.8)
Muscle %	43.8 (± 4.4, 31.1–49.6)
Preexisting medical conditions (Yes %)	48%
Medication (Yes %)	22%
Previous DCI (Yes %)	30%
Lifetime max. depth (meters)	109 (± 33, 60–225)
Lifetime number of dives	845 (± 413, 300–1950)
Experience of diving (yrs.)	14 (± 6, 6–30)
Diluent He (measured after dive)	19.3 (± 1.8, 16.2–24.1)
Diluent N_2_ (measured after dive)	40.0 (± 3.5, 27.1–45.1)

*Average, SD and range shown when applicable*.

The study was done in accordance with the Declaration of Helsinki. Ethical permission (HUS/976/2019) has been granted by the Ethical Committee of Helsinki University Hospital and a Research permit (HUS/44/2019, §21, 8.8.2019) was granted by the Helsinki University Hospital. In addition, every diver signed an informed consent form to participate in this study.

### Diving Equipment and Breathing Gas

All subjects used their own diving equipment, including their regular dry suit and undergarments. The subjects were allowed to wear an electrical heating vest under the dry suit, but the use of the vest was limited to emergency cases only, since the skin temperature was being measured during the dive, and the vest would influence the body temperature and would cause false readings for the temperature. Divers used CCR with a standardized diluent trimix of 20% helium and 40% nitrogen and the oxygen controllers maintained a constant oxygen partial pressure in the breathing loop (pO_2_ 70 kPa at the beginning of the dive and pO_2_ 120 kPa at the bottom depth and during ascent). The CCR units in this study were as follows: JJ CCR [number of divers (*n*) = 14], Megalodon (*n* = 1), rEVO (*n* = 4), AP inspiration evolution (*n* = 2), Sentinel (*n* = 1), and T-Reb (*n* = 1). These CCR devices all work according to the same principals and are similar in terms of function. Regardless of CCR device, all divers used the same set points in their device, which means that in practice all divers breathed the same gas composition during the dives.

### Critical Flicker Fusion Frequency Device and Measurement Procedure

**Figure 1:**
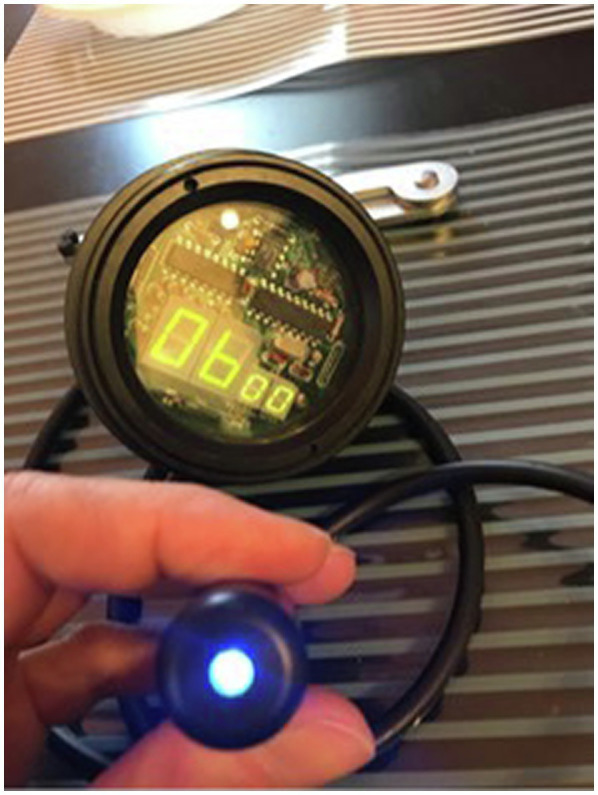
The critical flicker fusion frequency device that was used in the study to evaluate the divers’ level of alertness.

A water-resistant device specially produced for diving medicine research, courtesy of DAN Europe Diving Safety Laboratory, Roseto, Italy, was used for the assessment of the CFFF. The prototype device was built by the Human Breathing Technology (HBT Technology, Trieste, Italy) and consisted of a cylindrical water-resistant plastic housing with a rotating ring that was used to operate the machine. A flickering LED light that could be seen through a plastic housing at the end of a watertight cable was used for measuring the CFFF. This cable was attached to the device. An LCD screen is located in the center of the rotating ring, which shows a number indicating the frequency that the LED is flickering at. The device was attached to a D-ring on the divers’ harness with a bolt snap, and it was taken off during the measurements. The device is shown in Figure 1.

The divers were taught to operate the CFFF device and all subjects performed the test on one another in groups of three, with the same divers that they were planned to dive with. Diving gloves were used during training, so that differences in handling the device would not differ from when taking surface measurements and taking the measurements during the dives.

After training, two test performances were done in the way that one diver operated the CFFF device while one diver was assessed, the operator turned the ring clockwise to increase the frequency of the flickering LED light. When the test-performing diver no longer saw the LED light flickering but saw a stable light he/she signaled to the operator by lifting a hand and the operator then stopped the increase of the light by turning the ring counterclockwise by one stop. The operator then showed the result to a third diver who wrote the value on a piece of wet note paper. The divers then changed roles so that everyone was assessed twice.

The divers repeated the test two times during the dive at the bottom (45 meters) and again at the surface directly after ascent. All performances were done in the same groups of three and with the precisely same procedure.

### Subjective Warmth Assessment

The divers were also asked to assess their subjective feeling of warmth on a scale from 1 to 10, where 1 indicated being freezing cold, 5 being neutral, and 10 being burning hot. We asked the subjects to assess the warmth in three regions of their body, their hands, feet, and torsos. The divers assessed their warmth four times during the dive, after 30 min, 40 min, 50 min, and 60 min of immersion. They wrote the assessed scores in their wet notes.

### Objective Temperature Measurement

The divers’ skin temperature (Tskin) was measured and recorded with the ACR Smart Reader Plus 8-system (ACR Systems Inc., Vancouver, BC, Canada) at four standardized skin sites: the neck; scapula; hand; and shin, every 5 min throughout the dives (The International Organization for Standardization, 2004). Water temperature was monitored during the dives with the Suunto EON Core and the Suunto D5 dive devices.

### Preparation and Diving Procedure

The tests were done in January over the course of two weekends in a water-filled, thin ice-covered quarry at Ojamo in Lohja, Finland. The water temperature was 2°C at the decompression/safety stop and 4°C at the bottom. The divers were briefed on the diving protocol and safety issues as they arrived at the diving site. Their diving gear was prepared and the preliminary CFFF measurements were taken indoors at constant room temperature near the ice hole in which the divers later submerged. The divers were instructed not to use alcohol for 24 h before the tests. On the day of the dive, they were told to hydrate according to their normal routines until 2 h before the dive, thereafter 5 dl of a sports drink (Gatorade, PepsiCo, Nordic Finland Ltd., Helsinki, Finland) was consumed. To study the demographics of the diver population, the body mass index (BMI), fat % and muscle % were measured with an InBody 720 composition analyzer (Biospace Ltd., Seoul, South-Korea). These are described in Table 1.

The divers did the first set of CFFF tests at the surface as discussed earlier in the methods section. To ensure identical dives, a pre-set line from the surface to the target depth of 45 meters was set. The dive plan was instructed to all divers and all divers followed it successfully resulting in a maximal depth of 45.5 ± 0.7 meters, dive time of 67.4 ± 3.9 min. Temperatures were ranging from 4.3°C in the bottom to 3.1°C on decompression stop indicating reverse thermocline typical in winter/arctic conditions. Water temperature was measured by the dive computer. The dive plan was as follows: immersion with face out of water (5 min), face in the water at the surface (5 min), and start of dive and submersion to a 6 meters depth (5 min). After this, the divers followed the pre-set line down the slope to the depth of 45 meters. At the bottom depth, the divers spent approximately 18 min and performed the CFFF tests. Decompression was done with the following profile: Suunto Fused RGBM 2 with personal adjustment + 2 (Suunto EON Core and Suunto D5 dive computers). Immediately after surfacing, the divers took the final set of CFFF tests. Thus, the measurements were taken three times: before the dive at the surface, during the dive at the target depth (45 meters), and a third measurement at the surface after the dive.

## Statistics

A statistical analysis of the CFFF and subjective warmth data were performed with JMP^®^, Version 15.1.0 (SAS Institute Inc., Cary, NC, 1989–2019). All continuous measurement outcomes passed the Shapiro-Wilk test allowing us to assume a normal distribution and thus use a one sample *t*-test for analysis. […] The analyses were performed to the mean taken from both CFFF measurements in each time point. We excluded three bottom CFFF measurements and five of the post-dive CFFF measurements due to unclear readings. The cause was found to be insufficient voltage of the batteries. For these reasons, 20 measurements were used for the pre-dive and during-dive statistical analysis and 17 measurements were used for pre-dive and post-dive analysis. After normalizing the pre-dive measurements to 100%, the relative change in CFFF was calculated as a percentage from the baseline measurement at each time point. This allowed us to consider the magnitude of change instead of absolute values. Therefore, each diver acted as his/her own control in CFFF measurements. We also excluded one diver’s subjective warmth assessments in the 30, 40, 50, and 60 min time points and two more were excluded in the 60 min time point due to missing reporting.

Data with ordinal scales were analyzed using the Bowker’s test since Wilcoxon/Kruskal-Wallis test was not applicable to this case. The feeling of cold was measured from the same subjects in different time points and thus, the variables are dependent.

Values of *p* of 0.05 or below were considered statistically significant. All data are presented as means (± *SD*, range) when applicable, if not, they are presented as percentages.

For the temperature data, the values of *p* were calculated using the Satterthwaite’s method of approximating the degrees of freedom. The analyses were done with R version 4.0.4 using packages ggplot2 for visualizations and lme4 for the modeling.

The estimation of the decrease in temperature during the dive was made by noting the area-weighted Tskin between each diver. The Tskin was calculated using the ISO9886 weighting coefficients (The International Organization for Standardization, 2004).

## Results

### Diver Population and the Dive

Table 1 shows the demographics of the study population. The mean age of our population was 44.9 years. The mean fat percentage was 22.7% (± 7.7, 12.6–46.8) and the mean muscle percentage was 43.8 (± 4.4, 31.1–49.6). The mean BMI in our population was 27.6 (± 3.9, 23.4–39.8). 48% (11 divers) of the divers had preexisting medical conditions and 22% of our population had some medication for these conditions. The medical conditions in our population included arthrosis, allergies, asthma, hypertension, impingement, discus prolapse, celiac disease, obesity, migraine with aura, and treated supraventricular tachycardia. None of these medical conditions were considered to be a contraindication for diving. 30% of the subjects had a case of previous DCI and the mean diving experience in years was 14 (± 6, 6–30). Mean depth of the research dive was 45.5 ± 0.7 meters, and the dive time was 67.4 ± 3.9 min. Water temperatures were 3.1–4.3°C.

### Critical Flicker Fusion Frequency

**Figure 2:**
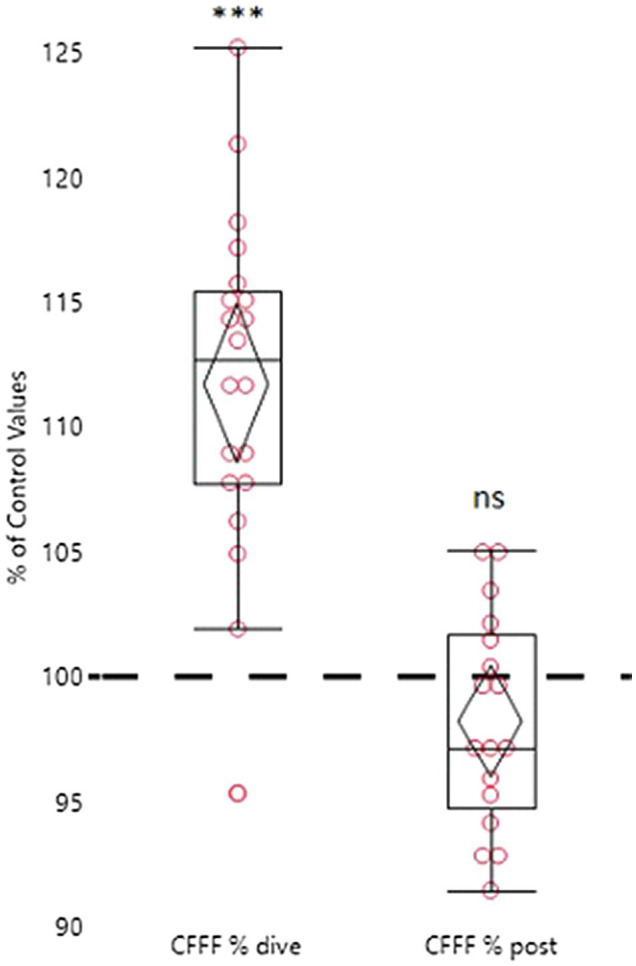
Percentage variation of CFFF during and after the dive to 45 meters with CCR (low set point 0.7 and high set point 1.2, the diluent being trimix 19/40) in relation to pre-dive measurements. The pre-dive measurements are marked as the 100% reference line. Each subject is compared to his or her own measurements. The box plot represents quantiles, and the diamond represents the 95% confidence interval (^***^p = < 0.0001, ns = not significant). Sample size is 20 in the pre-dive analysis and 17 in the pre-post analysis.

The change of CFFF values is shown in Figure 2. Compared to the pre-dive measurements, there was a statistically significant increase in the CFFF readings when the subjects reached the target depth of 111.7% (± 6.8108.5–114.9%; *p* < 0.0001) of the control values. The post-dive values showed non-significant differences compared to the baseline values, at 98.2% (± 4.3%, 91.4–105.0%; *p = ns*).

### Subjective Warmth Assessment

**Figure 3:**
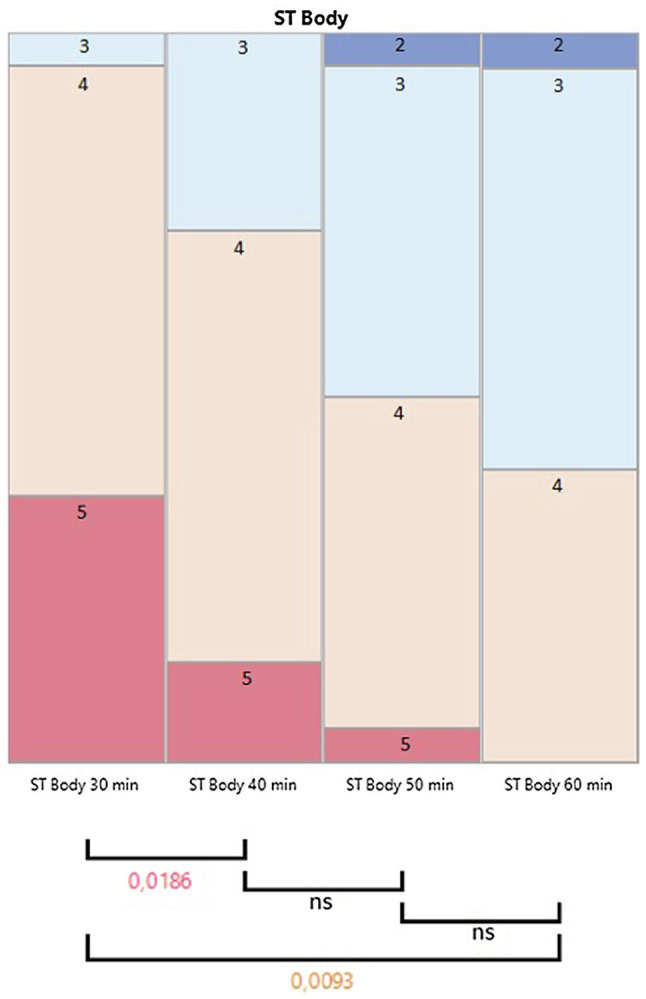
The subjective warmth assessment for four time points at 30, 40, 50, and 60 min after the beginning of the dive. The scale is from 5 to 1, where 5 stands for neutral warmth and 1 for freezing cold. The boxes represent the percentage of answers in each category. There is a statistically significant drop in the “5” category and a statistically significant increase in the “3” category. The sample size is 22 in the ST body 30 min, ST body 40 min and ST body 50 min categories, and 20 in the ST body 60 min category.

The subjective warmth assessment results are shown in Figure 3. There is a statistically significant difference between the 30-min and 40-min assessments (*p* = 0.02) and between the 30-min and 60-min assessments (*p* = 0.01). A significant difference was not found between the 40-min and 50-min (*p = ns*) or 50-min and 60-min assessments (*p = ns*). We did not find a statistically significant correlation between the BMI or fat percent and a change in the subjective temperature assessment (*p = ns*).

### Objective Warmth Measurements

The linear mixed model estimated the Tskin to drop by 0.48°C per 10 min during the dive (0.43–0.52 95% CI, *p* < 0.001).

**Figure 4:**
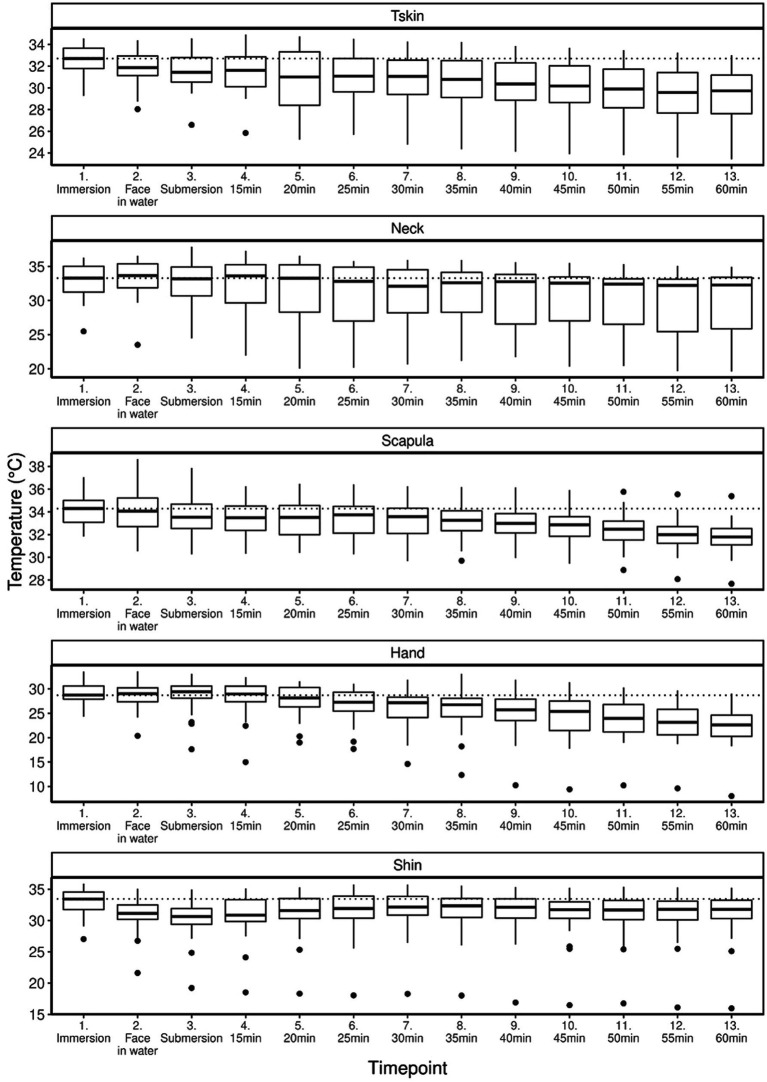
The linear regression model of temperature measurements from immersion, when the divers put their faces into the water, when the divers submersed and from that point onward in 5-min intervals. There was a statistically significant (*p* < 0.001) drop in temperature in all locations of measurement.

As shown in Figure 4, for the Tneck values, the same model estimated a drop of 0.63°C per 10 min (0.53–0.73 95% CI, *p* < 0.001); for the Tscapula values, the model estimated a 0.36°C drop per 10 min (0.33–0.40 95% CI, *p* < 0.001); for the Thand values, the model estimated a 1.15°C (1.07–1.24 95% CI, *p* < 0.001); and for the Tshin values, the model estimated a 0.09°C for 10 min, respectively (0.03–0.15 95°C, *p* < 0.001).

## Discussion

To our knowledge, this is the first study where CFFF is measured on a large group of CCR divers in open-water Arctic conditions. Moreover, this is the first study where the influence of measured skin temperature on the CFFF results is assessed in divers.

In our study, we showed a significant drop in skin temperature as well as a subjective decrease in thermal comfort in the divers. This mild change did not have an effect on diver’s alertness or cognitive performance. Divers used a CCR device that allowed them to breath circulating pre-warmed gas. Therefore, heat loss may have been less than when using an open circuit diving system where heat is lost with every breath. However, we cannot exclude the possibility that greater heat loss could have had an effect on diver’s alertness, and therefore potentially jeopardize diver’s safety.

Our study was conducted in water temperatures ranging from 3.1 to 4.3°C. But even though the temperature differed a lot from earlier studies, the CFFF profile was in line with results from the previous studies (Seki and Hugon, 1976; Balestra et al., 2012; Hemelryck et al., 2013; Kot et al., 2015; Lafère et al., 2016, 2019; Tikkinen et al., 2016; Rocco et al., 2019; Vrijdag et al., 2020), which would indicate that at least in this study setting, a cold environment and a decreased in skin temperature were not major factors to influence the CFFF profile. The increase in CFFF results at diving depth was very similar to the two studies conducted in otherwise similar study settings—in one earlier study, the increase in CFFF was in fact 111% compared to the pre-dive control values (Rocco et al., 2019; Vrijdag et al., 2020), which is very similar to our study results (111, 7%). Even though our subjects wore dry suits, the divers experienced subjective cold during the dives (Figure 3) and their skin temperature decreased significantly (Figure 4).

In our study, the CFFF values increased during the dives, and as the divers surfaced, the CFFF values were normalized to the same level as they were before the dive or even to a slightly depressed level, although this was not statistically significant. This post-dive depression of the CFFF has been noted in other studies before (Balestra et al., 2012) but is still a relatively newly described finding and is not fully understood yet. In this aforementioned study, there was a significant depression of CFFF values for at least 30 min after surfacing. This requires more attention in our opinion and possibly modifications to diving guidelines, since the subjective feelings of narcosis are essentially nonexistent but there is objective evidence of cerebral impairment. This might indeed be a problem in situations requiring fast and accurate judgment, especially in military or industrial diving.

The dives in our study were also much longer than the dives in other studies [60 min compared to 5–25 min in other studies (Seki and Hugon, 1976; Balestra et al., 2012; Hemelryck et al., 2013; Kot et al., 2015; Lafère et al., 2016, 2019; Tikkinen et al., 2016; Rocco et al., 2019; Vrijdag et al., 2020)]. The effect of this in regard to CFFF is unclear; however, since according to the lipid theory (Jibu, 2001; Wlodarczyk et al., 2006), it has been advised that in case of nitrogen narcosis, a diver should ascend a few meters for the narcosis effect to dissipate, which would mean that the amount of narcosis is dependent of the depth, not the length of the dive. The persisting CFFF depression might have affected our results too, but the amount of this persisting depression is, however, unclear. Since our dive was longer than in other studies, there was a substantially higher nitrogen load (our dive being a decompression dive) but the extent to which this affects our results is debatable.

Closed circuit rebreather diving with a trimix (19/40) did not have a greater influence on the CFFF profile compared to other studies with compressed air. The findings concerning breathing gases and CFFF have varied in other studies, but taking into account the composition of these gases, some deductions can be made. In one study by Rocco et al. (2019), one of the groups used a gas quite similar to ours their gas being trimix 21/35 and our being trimix 19/40. Of course, the gases are not fully comparable, but the amount of nitrogen in these gases was similar (they had 44% and we had 40% N_2_), and not surprisingly, the CFFF values were similar too. The rough profile of the CFFF values is very similar compared to other studies too, which would indicate that helium in these quantities and depths does not have neuromodulatory effects, which is an expected finding due to the lipid theory (Jibu, 2001; Wlodarczyk et al., 2006). The amount of oxygen must be taken into account when comparing our study to others. It has been shown that even NBO increases nerve conduction, and this is likely to be related to ROS (Brerro-Saby et al., 2010). There has also been shown a dose-dependent effect of oxygen on neuronal excitability using CFFF (Kot et al., 2015). Furthermore, there is also a sedating, narcotic effect of oxygen which was hypothesized as early as in the 1970s (Thomas, 1974; Smith and Paton, 1976; Hesser et al., 1978), but has been widely accepted and proved only in recent years. Thus, it is hard to ascertain the extent of the effect that oxygen had in our study. It is undoubtedly a factor to be taken into account, but in our study setting, it is hard to differentiate between effects of different gases in our data.

Our subjects had a larger BMI than subjects in other studies (Balestra et al., 2012; Hemelryck et al., 2013; Lafère et al., 2016, 2019; Tikkinen et al., 2016; Rocco et al., 2019). In most of the studies conducted on divers, the subjects have usually been very fit due to the nature of the sport and strict criteria by which people are selected for the studies. This did not seem to influence the CFFF results. Additionally, there does not seem to be a correlation between the subjective sensation of cold and BMI in our population. This is counterintuitive but might be explained with our small sample size and unstandardized diving gear that the divers used. Since everyone used their own diving gear, those who tend to get cold more easily may have had thicker undergarments. There has, however, been noted a diminished thermogenesis response in overweight men during cooling which might explain our findings (Claessens-van Ooijen et al., 2006).

There are some limitations, albeit also strengths, in our study. Firstly, the number of measurements conducted for each diver in each time point is limited. It would have been very hard to facilitate numerous measurements in our study, as it would have significantly increased the dive time and therefore the decompression stress in this deep and cold dive. However, our study was planned to include only CCR divers to have an exceptional large cohort for this subgroup.

Secondly, because of the sex distribution of the technical diver population in Finland, we were only able recruited two female divers, but no earlier studies have shown significant differences in physiological responses for these stimuli between women and men. This could also be a strength of our study, since in many comparable studies (Kot et al., 2015; Lafère et al., 2016; Fernandez et al., 2019) there were solely male divers. Our study did not reveal differences in CFFF between female and female divers, although with only two females participating no conclusions can be made.

Thirdly, our results cannot be generalized to all divers, as we recruited only very experienced divers due to the demanding conditions and the requirement to dive with a CCR device. Hence, all beginners and recreational divers were excluded. However, the increasing popularity of technical diving, and especially CCR diving, among recreational divers in the last 10–15 years is an important reason to study more this subgroup of divers, as they tend to perform more demanding dives with higher risk profile, and therefore, cognitive functions play a critical role.

Fourth, there may have been a possibility that the learning could have influenced the CFFF results. The CFFF values elevated at the second time point, i.e., at arriving at the target depth. On the other hand, the CFFF values returned to the baseline when the divers surfaced, which would speak against the possibility of learning.

Fifth, the underwater CFFF measurement was taken in a different environment than the other two surface measurements. On the other hand, we estimate that this did not influence the results since the divers used gloves in all testing situations and the same testing protocol was conducted in all measurements. Additionally, it is unlikely that temperature would have influenced the subjects’ dexterity during the underwater CFFF. From the measured skin temperatures, we can see that the underwater measurements were taken at a time point at 25–30 min when all divers had a hand skin temperature within acceptable limits.

## Conclusion

Our findings indicate that a cold environment and a significant drop in skin temperature together with a decrease in subjective thermal comfort do not influence CFFF results on experienced CCR divers. Our study also suggests that the usage of CCR devices or trimix gases does not affect the general CFFF profile, or the hyper-alertness of oxygen, which has been seen in other comparable studies.

We hypothesize that a cold-water environment does not change the magnitude of cerebral impairment observed in diving. From that reason, we conclude that, at least in this study setting, CCR diving is safe from the perspective in alertness and cognitive performance. Cold water might, however, affect the ability to perform work which requires concentration or precise motor abilities for long term as the hypothermia increased toward end of the 60-min dive.

Complicated theories to describe changes in CFFF during diving have been suggested, and more research should still be done as the narcotic/excitatory effects of the gases breathed in commercial and technical diving are still not fully understood.

## Data Availability Statement

The raw data supporting the conclusions of this article will be made available by the authors, without undue reservation.

## Ethics Statement

The studies involving human participants were reviewed and approved by the Ethical Committee of Helsinki University Hospital (HUS/976/2019). The patients/participants provided their written informed consent to participate in this study.

## Author Contributions

WP, RL, LT, and AR-S planned the study and participated in the data gathering and processing. WP and RL were the main writers of the manuscript and participated in data analysis. LT and AR-S participated in the writing of the manuscript. All authors contributed to the article and approved the submitted version.

## Conflict of Interest

The authors declare that the research was conducted in the absence of any commercial or financial relationships that could be construed as a potential conflict of interest.

## Publisher’s Note

All claims expressed in this article are solely those of the authors and do not necessarily represent those of their affiliated organizations, or those of the publisher, the editors and the reviewers. Any product that may be evaluated in this article, or claim that may be made by its manufacturer, is not guaranteed or endorsed by the publisher.
